# Socio-Demographic Factors Influencing the Use of Assistive Technology among Children with Disabilities in Malawi

**DOI:** 10.3390/ijerph18063062

**Published:** 2021-03-16

**Authors:** Monica Jamali-Phiri, Ikenna D. Ebuenyi, Emma M. Smith, Juba Alyce Kafumba, Malcolm MacLachlan, Alister Munthali

**Affiliations:** 1 Centre for Social Research, University of Malawi, Zomba, Malawi; jkafumba@cc.ac.mw (J.A.K.); amunthali@cc.ac.mw (A.M.); 2 Assisting Living & Learning (ALL) Institute, Department of Psychology, Maynooth University, W23 F2K8 Maynooth, Ireland; ikenna.ebuenyi@mu.ie (I.D.E.); Emma.Smith@mu.ie (E.M.S.); Mac.MacLachlan@mu.ie (M.M.); 3 Olomouc University Social Health Institute (OUSHI), Palacký University, 779 00 Olomouc, Czech Republic

**Keywords:** Assistive Technology, disability, children, socio-demographic factors

## Abstract

This paper aims to address the information gap on the influence of socio-demographic factors on access and utilization of Assistive Technology (AT) among children with disabilities in Malawi. Thus, it contributes towards the realization of the recommendations of the UN Convention on the Rights of Persons with disabilities and the development of a framework for creating an effective national AT policy. The paper used two statistically matched datasets, namely, the 2017 survey on Living conditions among persons with disabilities in Malawi and the 2015-16 Malawi Demographic and Health survey. Logistic regression and structural equation modeling techniques were utilized to assess the influence of socio-demographic factors on the use of AT among children with disabilities. The results indicate that there is a high level of unmet need for AT among young children aged 2 to 9 and those living in urban areas. The results further indicate that children with multiple disabilities have lower odds (OR = 0.924) of using AT for personal mobility compared to children with a single functional difficulty. These results entail that AT needs for children with multiple disabilities are not adequately addressed. Therefore, when developing policies on AT, younger children and those with multiple disabilities need to be specifically targeted.

## 1. Introduction

Despite the global need and recognized benefits of assistive technology (AT), access to it in the sub-Saharan Africa region continues to be limited [1,2,3]. Assistive technology is an umbrella term, which encompasses systems and services related to the delivery of assistive products and services [3]. Assistive products are defined by the World Health Organization’s Global Cooperation on Assistive Technology (GATE) initiative as any product (including devices, equipment, instruments, and software) either specially designed or produced or generally available, whose primary purpose is to maintain or improve an individual’s functioning and independence and thereby promote their wellbeing [4]. Addressing this unmet need in the region is essential as AT is recognized as an important mediator in the equitable achievement of the Sustainable Development Goals [5] and the realization of the recommendations of the Convention on the Rights of Persons with Disabilities [3,6]. Individuals of all ages require access to AT; for children, AT is critical to their development and learning needs [3]. Assistive technology also enables children to make choices and to direct their own care with the use of augmented or alternative communication [7]. For example, children with disabilities in Kenya have been found to benefit from AT because it helps them to compete for limited academic or economic resources with their non-disabled counterparts [8]. In Namibia, constructive digital AT assists children with hearing impairment to attain mathematical skills [9]. Parents of children with physical, visual, and hearing disabilities in Ghana also perceive AT as beneficial as it enables their children to develop and actively participate in societal activities [7].

Although AT is beneficial for the development and participation of children in societies, research conducted in the Sub-Saharan region demonstrates that there is limited access to these technologies. The World Health Organization estimates that only 5% to 15% of AT needs are met in the African region [10]. Matter and Eide [11] found that 44% of persons with disabilities in Botswana who needed AT had not received it, whilst in Swaziland, 67% had an unmet need for AT. Several factors have been found to contribute to the limited availability of AT in the region. The factors include low prioritization of AT in national legislation, policies, or strategies [12]; the high cost of AT and rehabilitation services [11]; limited awareness of the benefits of using assistive devices and the services available to ensure access to them [12]; the negative attitude of the head-teachers, teachers, support staff, and students with and without disability [8]; social stigma and being singled-out as disabled through the use of AT [13].

In Malawi, only 4.5% of the total population of persons with disabilities have access to AT [14]. Amongst children aged 0 to 17, only 1.9% have access to AT. The AT being used in the country include mobility products (e.g., Wheelchairs, walking sticks, and white canes), information (e.g., eyeglasses and hearing aids), personal care (shower seats and safety rails), communication (sign language interpreter and portable writers, Braille), technologies for handling products and goods, household items, and computer assistive technologies [15,16]. Other AT include clubfoot braces, pressure relief cushions, standing frames, ramps, Rollators, and Therapeutic footwear [17]. A high proportion of the AT being used are sourced through donations or personal income (37.7%) and from government facilities (27.2%) [16].

As much as there is information on the proportional distribution of the use of AT among children with disabilities in Malawi, little is known about the factors that facilitate or hinder access or the use of AT among these children. This paper, therefore, aims to address this information gap by exploring socio-demographic factors such as age and wealth status that are likely to influence access and utilization of AT among children aged 2 to 17. The results obtained in this study form part of the participatory work for the larger Assistive Product List Implementation Creating Enablement of inclusive Sustainable Development Goals (APPLICABLE) project and will help to guide the development of a framework for creating an effective national AT (AT) policy [18].

## 2. Materials and Methods

### 2.1. Study Design

A secondary-data analysis was employed in this study, where data from two-nationally representative surveys, namely, the 2017 Living conditions among persons with disabilities (2017 LCS) and the 2015-16 Malawi Demographic and Health Survey (2015-16 MDHS) were utilized [19]. These two datasets involved larger samples that were representative of the target population and thus allowed for greater validity and generalization of the study findings. The 2017 LCS and the 2015-16 MDHS also contain substantial disability data that can be used in the development of new knowledge, however, researchers in this country always underutilize this data.

### 2.2. Sample Surveys

The data used to examine the relationship between socio-demographic factors and the use of AT was obtained by statistically matching the 2017 LCS and the 2015-16 MDHS [19]. The 2017 LCS is a nationally representative survey that was conducted between 2016 and 2017 among households of individuals with and without disabilities. This study was a follow-up to a similar study that was conducted between 2003 and 2004. The survey was conducted to map the living conditions among persons with disabilities and to compare the living conditions of the disabled with those of the non-disabled population. The survey collected information on disability indicators that would determine any changes in the living conditions of persons with disabilities [14]. For children with disabilities, their information was collected from either the head of the household in the absence of the child or the presence of the disabled child using the UNICEF module on Child Functioning [14].

Regarding the use of assistive technologies, the 2017 LCS survey participants with various functional limitations were asked if they were using the following assistive devices or products: for personal mobility (wheelchairs, crutches, walking sticks, white cane, guide dog, standing frame); for obtaining information (eyeglasses, hearing aids, magnifying glass, enlarged print, and braille); for personal care and protection (special fasteners, bath, and shower seats, toilet seat raiser, commode chairs, safety rails, and eating aids); for communication (sign language interpreter, fax, portable writer, and portable computers); for handling products and goods (gripping tongs, aids for opening containers, and tools for gardening); in-terms of household items (flashlight on the doorbell, amplified telephone, vibrating alarm clock); for computer assistive technologies (keyboard for the blind). The assistive technologies were not differentiated in terms of low cost, improvised aids, and manufactured aids [14]. Among the 1475 children with disabilities that were sampled in the survey, only 1.9% of the sample was using any type of assistive technology.

The 2015-16 MDHS, on the other hand, is a cross-sectional survey that was conducted from October 2015 to February 2016. This study was conducted by the Malawi National Statistics Office in collaboration with the Ministry of Health [20]. The survey was conducted to provide an overview of the country’s population, maternal and child health, and other health indicators including measures of nutritional status and knowledge and attitude of women and men about sexually transmitted diseases [20]. Regarding disability, the survey collected information about functional limitations or disabilities of children aged 2 to 17. This information was obtained from the respondents through a household questionnaire. The questions on disability or functional limitation included speech and language, hearing, vision, learning (cognition and intellectual development) mobility and motor skills, emotions, and behaviors [20]. Concerning assistive devices, the survey only asked about the use glasses and hearing aids.

## 3. Study Variables

### 3.1. Dependent Variable

#### 3.1.1. Use of Assistive Technology

The use of AT is the dependent variable of this study. During the 2017 LCS, participants in the study were asked if they use any assistive device (product). The question included the use of an assistive product for personal care and protection; personal mobility; handling products and goods, household items, computer AT, communication, and information. Responses to this question included a yes or no response. These responses have been recorded into a binary response of 1 and 0 so that a binary logistic regression model could be performed to determine the probability of responding to a yes or no depending on one’s socio-demographic characteristics.

#### 3.1.2. Use of an Assistive Product for Personal Mobility

Further to the question of the use of an assistive product, the survey participants in the 2017 LCS survey were asked to describe the type of assistive product that they were using. The responses to this question included assistive products for information, communication, personal mobility, household items, personal care, and protection, for handling products and goods, computer assistive technologies, and other types of devices. This study focuses only on assistive products for personal mobility because there were few cases of children who were using other types of assistive products. Thus, the likelihood of using such products could not be estimated using logistic regression models.

### 3.2. Independent Variables

Several socio-demographic factors such as age, gender, and educational status influence access and utilization of AT among persons with disabilities. This study focuses on the influence of family structure (male or female-headed households), household income, age, sex, place of residence, region and educational status, severity, and type of disability.

The disability variable used in this study was derived from the 2015-16 Malawi Demographic and Health survey. During the survey, household informants were asked to evaluate the health state of children aged 2 to 9 with regards to delay in sitting, standing, or walking, difficulty seeing, difficulty hearing, difficulty understanding what has been said, difficulty moving arms and legs, having fits or losing consciousness, difficulty in learning to do things their age, speaking any recognizable words or speech being different from the rest of the family members, or appear to be mentally backward, dull, or slow. (Note: the terms mentally backward, dull, or slow are the terms that were used during the 2015-16 DHS survey and were agreed upon by the participating Disabled People’s Organizations.). The household informants also gave information on the functional status of children aged 10 to 17. This information pertained to their difficulty in seeing even if wearing glasses or contact lenses, difficulty hearing even if wearing hearing aids, communication difficulty, difficulty in remembering or concentrating, difficulty walking, washing or dressing. Responses to the health states questions were in a binary format of yes or no. The responses were then summed up by the researcher to create a composite score or functioning or disability score measuring from 0 to 9. A summary of these independent variables is presented in Table 1.

### 3.3. Analysis

To achieve the objective of this paper, the 2017 LCS and the 2016-17 MDHS were statistically matched. Statistical matching is a technique used by practitioners to combine information from distinct data sources referring to the same target population [21,22]. The technique involves two data files, A and B, where A and B share a set of common variables (*X)*, with variables *Y* observed only in A and variables *Z* observed only in B. The purpose of carrying out statistical matching is to estimate the correlation coefficient between *Y* and *Z* conditional on *X* variables at a macro level [22]. In some cases, statistical matching is used to create a synthetic data source in which all the variables X, Y, and *Z* are available— the micro case [21,23].

The 2017 LCS and the 2015-16 MDHS datasets were statistically matched in this paper due to the limited availability of data on access and utilization of assistive devices among children with disabilities in the 2015-16 MDHS survey. In statistically matching the two datasets, it was assumed that the surveys are distinct but were collected from the same population in Malawi [21,24]. Statistical matching of the datasets was also based on the assumption that there exists no auxiliary information on the statistical relationship between disability and use of assistive devices (variables used in the matching process). This means that the statistical matching of the two datasets was performed under the *Conditional Independence Assumption* (CIA) [25]. The matching procedure was also performed with a view of exploring uncertainty due to the absence of joint information on the disability index and the use of assistive devices.

The statistical matching of the 2017 LCS and the 2015-16 MDHS involved the harmonization of the common variables in the two datasets to facilitate the coherence of the datasets. Thus, the value labels of variables such as sex, place of residence, and level of education, and age groups were given the same value labels. Apart from harmonization, the two datasets were also adjusted for missing values. This was achieved by removing irrelevant values such as people aged 18 and above and those aged less than 2. In addition to adjusting for missing values, and sample populations, frequency analysis of the common variables was also carried out to determine the marginal distribution of the imputed values. Even though the two datasets had common variables, further analysis was conducted to identify variables that significantly explain the variation in the target variables that in this case were the use of assistive technologies and disability [25]. The analysis involved the use of the chi-square test and uncertainty measures of association. The statistical matching technique known as the random hot deck was then used to combine the two datasets so that a new dataset with imputed AT variables can be created. The accuracy of the statistical matching results was assessed using similarity or dissimilarity measures such as total variation distance, Hellinger’s distance, and Bhattacharyya coefficient [25]. The statistical matching procedure results were presented in another manuscript [19]. The results indicate that the number of children using any type of AT in the 2015-16 MDHS is 1.9% and those using mobility products or devices was 72.4% of the 1.9%.

The link between socio-demographic factors and access or utilization of AT has been analyzed using structural equation modeling and Logistic regression. In the regression model, the number of children using any type of AT was 249. Concerning the use of mobility products or devices, the number of children aged 2 to 17 was 180. Tests of associations, namely Chi-square tests, Logistic regression model, and Structure equation modeling have been used in this paper to examine the association between the use of AT and socio-demographic factors. A Chi-square test was used to test for the linear relationship between the dependent and independent variables. Logistic regression and structural equation modeling regressions were used to model the probability of using AT, given the socio-demographic characteristics of the child with a disability. All statistical analyses were performed using STATA version 15 [26,27].

## 4. Results

### 4.1. Demographic Characteristics of Children by Type of Disability

The demographic characteristics of the children have been separately presented because when measuring disability during the survey separate questions were used for children aged 2 to 9 and those aged 10 to 17. Separate questions or measures were used due to differences in developmental attainment.

The demographic characteristics presented in Table 2 indicate that a high proportion of children aged 2 to 9 in the country experienced difficulties in intellectual development (mentally backward, dull, or slow-6.9%) followed by hearing and speech difficulties (5.2% and 5.1%, respectively). The results also indicate that there is an almost equal proportional distribution of functional limitations or disabilities among male and female children. However, more than 80% of disabled children live in rural areas and a significant proportion is in primary school.

Table 3 further presents the demographic characteristics of children aged 10 to 17 with different types of disabilities. The results in the table indicate that the main functional limitation among this age group is difficulty in remembering or concentration (5.2%) followed by hearing difficulty (4.6%) and seeing (2.4%). Comparing with the younger age group (2 to 9), it is apparent that they both have cognitive, intellectual, and hearing difficulties; however, the younger age group has a high proportion of speech difficulties compared to the older group (5.1% versus 2.1%). Just as for children aged 2 to 9, a high proportion of children with disabilities in the older age group (10–17) live in rural areas and are in primary school.

### 4.2. Logistic Regression Results

**Figure 1:**
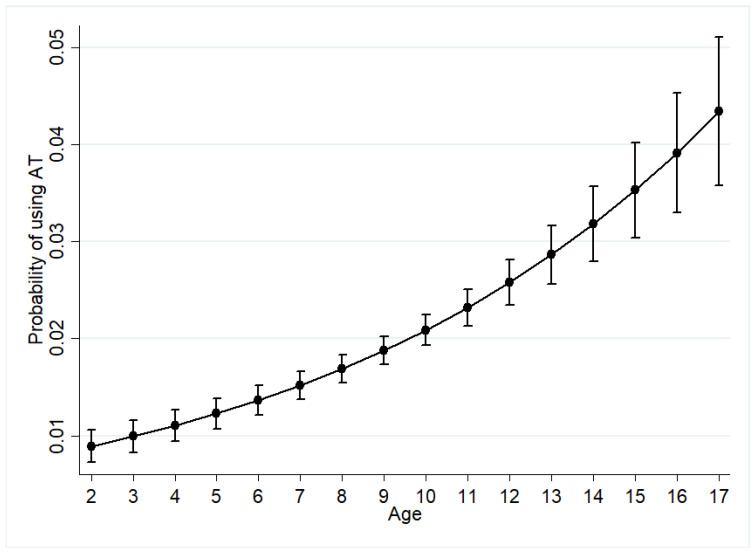
Probability of using assistive technology by age.

The logistic regression results demonstrate that the presence of impairment—whether walking, sitting, or mental development—is significantly associated with the use of AT among children with disabilities in the country. As the level of co-occurring impairment increases from 1 to 2 to 3, the odds of using an AT increase by 5% (Odds Ratio = 1.05) while holding age, sex, level of education, wealth status, and place of residence constant. Concerning the socio-demographic variables, only age and place of residence were found to be significantly associated with the use of any type of AT. As demonstrated in Figure 1 below, a unit increase in age increases the likelihood of using AT (Odd Ratio = 1.06) while controlling for all other independent variables in the model. Thus, older children are more likely to use any type of AT compared to younger children.

**Figure 2:**
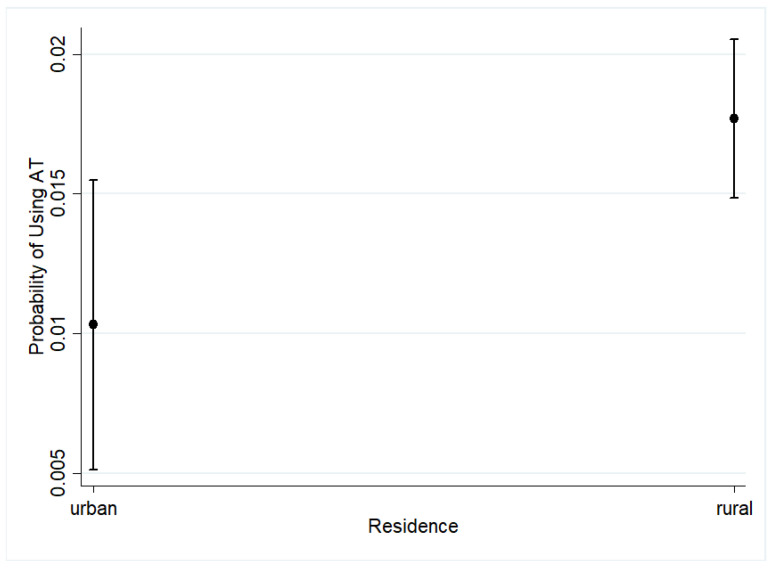
Probability of using assistive technology by place of residence.

The logistic regression results in Figure 2 further demonstrate that disabled children who live in rural areas have an increased likelihood of using AT compared to those living in urban areas with an estimated odds ratio of (OR = 1.73) at the 95% level of significance.

### 4.3. Use of AT for Personal Mobility as a Dependent Variable

Further to the modeling of the use of any AT, the study also investigated the use of AT for personal mobility. The logistic regression indicates that children with co-occurring impairments (i.e., 2 or more functional difficulties) have lower odds (Odds Ratio = 0.924) of using personal mobility devices compared to children with a single functional difficulty. Concerning socio-demographic variables, only place of residence significantly predicts the use of personal mobility devices among children with disabilities. Children with disabilities in rural areas are more likely to use AT for personal mobility compared to children with disabilities in urban areas.

### 4.4. Structure Equation Modeling Results

In addition to the assessment of the influence of socio-demographic variables on the use of any AT and the use of assistive technology for personal mobility, we also assessed the pathways through which the socio-demographic variables influence the use of assistive technologies among the children. Both estimated paths for the indirect effect were statistically significant (that is from socio-economic status to disability to use of assistive technology and from demographic characteristics to disability to use of assistive technology). The direct effect from socio-economic status to use of assistive technology and from demographic characteristics to use of assistive technology was also statistically significant at the significant value of 95%. Nonetheless, even though the model explained 45% (R-squared = 0.45) of the variation in the use of AT, both values for the direct and indirect effect were very minimal. While these results suggest that socio-demographic factors do negatively mediate the use of AT among children with disabilities, the mediating effect is minimal. This may suggest that there are other factors such as policy, environmental or attitudinal factors that influence the use of AT among these children.

## 5. Discussion

Results obtained from the assessment of the influence of socio-demographic factors on the use of AT among children with disabilities in this paper have indicated that age of the child and place of residence influence the use of any AT in Malawi. Concerning the use of AT for personal mobility, the results have demonstrated that only place of residence influences the use of these assistive products. The finding that a unit increase in age increases the likelihood of using any AT corresponds to the finding of Kaye, Yeager, and Reed [28] in their study of “disparities in usage of assistive technology among people with disabilities”. Kaye, Yeager, and Reed [28] also found the use of AT to increase markedly with age. According to Kaye, Yeager, and Reed [28], older people have increased use of AT because they have a greater need for it. With regards to the current study, older children (especially those aged 9 to 17) are more likely to use assistive technologies compared to young children (2 to 8) because they are either in school or have developed skills to manage the AT [10,16]. Higher usage of AT among older children could also relate to the perception that younger children may not attain important developmental skills if they become reliant on assistive products [2]. Nonetheless, the use of assistive products may also facilitate children in attaining developmental milestones.

Our findings regarding the influence of place of residence on both uses of any AT and use of AT for personal mobility do not correspond to the finding of Matter, and Eide [11] in two Southern African Countries (i.e., Botswana and Swaziland). Matter, and Eide [11] found a location (urban or rural) not to be significantly associated with the use of AT. However, the finding of the current study is supported by previous studies, which found the place of residence or location of a person with a disability to influence the use of AT [16,29]. For example, Visage, Eide, and Mannan [16] in their study of “a description of assistive technology sources, services and outcomes of use in several African settings” found walking mobility devices to be more commonly used in rural areas. Concerning the use of mobility products in rural areas, Zimmer and Chappell [29] explain that both environmental and attitudinal factors could be the facilitators for increased usage of mobility products. According to Zimmer and Chappell [29], urban residents may have negative attitudes towards the use of mobility products, thus making persons with disabilities shy away from using them. The availability of services such as health facilities and transport may also make a person with a disability less reliant on a mobility device. The observed disparities in use of AT among children with disabilities in rural and urban areas in this paper may be due to the variation in sample size between the 2017 LCS and the 2015-16 MDHS. The 2017 LCS (1475) had a small sample of children with disabilities aged 2 to 17 compared to the 2015-16 MDHS (13,121). Although we conjecture that it is related to socioeconomic factors as previously reported [16,29], we wish to further explore this possibility in future studies.

In addition to age and place of residence, the logistic regression results also indicated that having two or more functional disabilities is associated with the increased use of any assistive technology but a decreased use of assistive technology for personal mobility. This finding corresponds to previous studies, which have also found severity and type of disability to be significantly associated with the use of assistive technologies [11,28,30]. For example, Yeung, Lin, and Teng [30] in their study of the use of assistive products among individuals with disabilities in Taiwan found the use of assistive devices to significantly increase with increased severity of the disability. Nonetheless, some studies, such as the one by Matter, Harnis, Oderud [31] and Kaye, Yeager, and Reed [28], have found the association between use of AT and disability to be more influenced by the type of impairment, such as physical or intellectual, rather than severity of the functional limitation. Concerning the current study, there were limited cases of particular types of disabilities since only 2% of children with disabilities in the country use assistive technologies. The limited number of cases made it difficult to model the use of AT by a specific type of disability. With regards to decreased use of AT for personal mobility, as the level of co-occurring impairments increases, Lersilp, Putthinoi, and Lersilp [32] found nursery to grade 12 students with special needs (particularly those with hearing and visual disabilities) not to perceive assistive products as facilitators for mobility or use of school building. Since the descriptive results have indicated that a high proportion of children have either cognitive (remembering or concentrating) or hearing difficulties, this may explain why co-occurring impairments reduce the likelihood of using AT for personal mobility. Nonetheless, further research has to be conducted to understand why children with co-occurring impairments are less likely to use AT for personal mobility.

Further to the assessment of the influence of socio-demographic factors on the use of AT, the study also examined the mediating role of the socio-demographic factors in the relationship between the presence of a disability and the use of AT. The results of the mediating role have demonstrated that the role of socio-demographic factors is minimal, even though the overall model explains 45% of the variation in the use of AT. The minimal role of the socio-demographic variables could be related to the influence of other mediating factors such as societal attitudes, policy environment, and the accessibility of assistive technologies. Research on barriers to the use of AT among children with disabilities has indicated that the attitude of parents and teachers on the use of AT by children with disabilities affects the children’s use of AT both at home and in school [7,33]. For example, Osam, Opoku, Dogbe [7] found some parents of children with disabilities in Ghana not wanting their children to use AT because they thought that their children will not be empowered and that they would not be accepted by society, thus influencing the use of AT among disabled children.

In summary, the findings of this study indicate that socio-demographic variables such as age and place of residence do influence the use of AT among children with disabilities. However, the influence is minimal due to other external factors such as parental attitude or policy environment. This illustrates the importance of adopting a systemic approach to AT provision, where broader contextual factors are also taken into account [34]. For AT use to be accepted by children, their parents, and the social context in which they live, there is a need to explicitly promote positive images of AT use, that counter stigma that may be associated with disability or the AT used by children with disability.

The main limitation of this study is the sample size of children with disabilities using any type of assistive devices. In the 2017 LCS survey, only 1.9% of the 1475 children with disabilities were using any type of assistive technology. When this sample was matched with the 2015-16 MDHS, only 249 children with disabilities were using any type of assistive technology, thus limiting the sample of children with disabilities using any type of assistive technology in the 2015-16 MDHS. The small sample size of the 2017 LCS also limited the number of common variables that could be used for matching the two datasets. In the two datasets, the common variables include age, place of residence, sex, and level of education. However, place of residence and age were the only variables that could be used. The other limitation of this paper is that when collecting data on use of assistive technologies, the 2017 LCS did not differentiate between improvised technologies and manufactured technologies. This information could have helped in clarifying the increase in the use of AT in rural communities. Nonetheless, the 2017 LCS is amongst the most reliable disability survey that contains representative information on assistive technologies. Further to the lack of differentiation, qualitative information from in-depth interviews and focus group discussion could also have helped in explaining the challenges that children with disabilities experience when accessing assistive technologies. However, this was beyond the current paper as it focused on secondary data analysis.

## 6. Conclusions

This study investigated socio-demographic factors that influence the use of AT among children with disabilities using logistic regression and structural equation modeling. The results from the regression analysis have indicated that older children aged 10 and 17 with disabilities and disabled children living in rural areas utilize assistive technologies largely. Rural children with disabilities are also more likely to use assistive technologies for personal mobility, compared to urban children. These findings imply that the unmet need for AT, particularly that related to personal mobility such as wheelchairs and white canes among young children with disabilities, especially those aged 2 to 9 and those living in urban areas, are not being adequately addressed. This is likely to affect their educational and social development. Thus, there is a need to ensure that relevant policies and programs are developed to assists this population group so that they are not left behind.

This study has also shown that the mediating effect of socio-demographic factors on the use of assistive technologies among children with disabilities aged 2 to 17 in Malawi is negligible. This implies that the use of AT among these children is less related to their personal factors and more to other contextual factors such as the built environment, societal and political factors. Therefore, to ensure that the unmet need for AT, particularly that related to personal mobility for children with disabilities has been met, policymakers and service providers need to take a deliberately inclusive approach to policymaking [35] by addressing how the built and the political and social environment influence children with disabilities and their use of AT.

## Figures and Tables

**Table 1:** Table presenting a summary of socio-demographic variables used in the analysis.

Dependent Variable	Data Source
Ever used an assistive technology	2017 LCS
Use of mobility devices (wheelchairs, crutches, walking sticks, white cane, standing frame) (Note on mobility products or devices, the 2017 LCS did not differentiate the products by low cost, improvised aids, and manufactured aids.)	2017 LCS
**Independent Variable**	
Disability or functional limitation	2015-16 MDHS
Age	2015-16 MDHS
Sex	2015-16 MDHS
Place of residence	2015-16 MDHS
Region	2015-16 MDHS
Level of education	2015-16 MDHS
Household income (wealth status)	2015-16MDHS
Sex of the household head	2015-16 MDHS

**Table 2:** Percentage of children aged 2 to 9 with reported functioning problems or disability according to selected background characteristics.

Background Characteristics ^1^	Delay in Sitting Standing or Walking	Seeing Difficulty	Hearing Difficulty	Walking Difficulty	Has Fits or Lose Consciousness	Learning Difficulty	Speech Different from Normal	Mentally Backward or Slow ^2^	Number
Age group									
2–4	4.2	2.0	2.9	2.0	4.0	4.7	6.2	5.7	10,974
5–9	4.2	2.8	6.4	2.6	3.6	4.7	4.7	7.7	19,904
Sex									
Male	4.5	2.5	5.2	2.5	3.8	4.9	5.6	7.4	15,280
Female	4.0	2.5	5.1	2.3	3.6	4.5	4.6	6.5	15,598
Place of residence									
Urban	3.2	2.9	3.6	1.8	2.1	4.3	4.6	5.3	4722
Rural	4.4	2.5	5.4	2.5	4.0	4.8	5.2	7.2	26,156
Region									
Northern	3.3	2.3	4.0	1.8	3.0	3.5	4.5	3.6	6133
Central	4.4	2.8	5.3	2.2	5.1	4.8	4.4	7.3	10,134
Southern	4.5	2.4	5.6	2.7	3.1	5.1	5.9	8.1	14,611
Level of Education									
No education	4.5	2.2	3.7	2.4	4.2	5.0	6.4	6.2	14,679
Primary	4.1	2.8	6.5	2.4	3.2	4.4	4.3	7.6	16,188
Total	4.2	2.5	5.2	2.4	3.7	4.7	5.1	6.9	30,878

Note: ^1^ Note on Background characteristics, particularly wealth status and sex of the head of the household for ages 2 to 9, these variables have not been presented here but are presented in supplementary document, ^2^ Mentally backward or slow is the language that was used in the MDHS survey.

**Table 3:** Percentage of children aged 10–17 with reported functioning problems or disabilities according to selected background characteristics: 2015-16 Malawi Demographic and Health Survey (MDHS 2015-16).

Background Characteristics	Seeing Difficulty	Hearing Difficulty	Communication Difficulty	Difficulty Remembering/ Concentrating	Walking Difficulty	Washing/Dressing Difficulty	Number of Children
Age group							
10–14	2.2	4.6	2.0	5.1	1.2	1.8	19,116
15–17	2.8	4.6	2.1	6.3	1.3	1.2	7610
Total	2.4	4.6	2.1	5.4	1.2	1.7	26,726
Sex							
Male	2.1	4.4	2.3	5.4	1.2	1.9	13,712
Female	2.6	4.7	1.8	5.5	1.3	1.4	13,014
Total	2.4	4.6	2.1	5.4	1.2	1.7	26,726
Place of residence							
Urban	3.3	3.9	1.7	5.1	1.0	1.1	423
Rural	2.2	4.7	2.1	5.5	1.3	1.8	22,303
Total	2.4	4.6	2.1	5.4	1.2	1.7	26,726
Region							
Northern	2.2	4.0	1.5	3.1	1.3	1.6	5371
Central	2.4	4.4	2.0	6.2	1.3	1.4	8670
Southern	2.4	5.0	2.4	5.9	1.2	1.8	12,685
Total	2.4	4.6	2.1	5.4	1.2	1.7	26,726
Level of Education							
No education, Preschool	2.2	4.4	12.5	13.1	8.5	13.5	458
Primary	2.2	4.7	1.9	5.4	1.1	1.5	24,312
Secondary/higher	4.2	3.4	1.2	4.8	0.8	0.4	1956
Total	2.4	4.6	2.1	5.4	1.2	1.7	26,726
Wealth status							
poorest	2.0	4.8	2.6	5.4	1.5	1.8	4582
poorer	2.1	5.3	2.6	5.8	1.5	2.3	4699
middle	2.2	4.5	1.7	6.0	1.3	1.7	5369
richer	2.5	4.5	2.1	5.4	1.2	1.6	5993
richest	2.9	4.0	1.6	4.7	0.9	1.0	6083
Total	2.4	4.6	2.1	5.4	1.2	1.7	26,726
Sex of household head							
Male	2.3	4.5	1.9	5.4	1.1	1.5	18,080
Female	2.6	4.8	2.5	5.6	1.5	2.0	8646
Total	2.4	4.6	2.1	5.4	1.2	1.7	26,726

## Data Availability

The data supporting the reported results are available from the corresponding author on reasonable request.

## Supplementary Materials

The following are available online at https://www.mdpi.com/1660-4601/18/6/3062/s1.
