# Supplementary material for: Socio-Demographic Factors Influencing the Use of Assistive Technology among Children with Disabilities in Malawi
Source: Int J Environ Res Public Health. 2021 Mar 16;18(6):3062. doi: 10.3390/ijerph18063062 (PMC8002356; doi:10.3390/ijerph18063062)
Supplement: Supplementary file 1 [file ijerph-18-03062-s001.pdf]

Supplementary tables

Supplementary 1: Background characteristics of children aged 2 to 9 by type of disability or functional limitation (2015-16 MDHS)

Background characteristics	*Delay in sitting standing or walking*	*Seeing difficulty*	*Hearing difficulty*	*walking difficulty*	*Has fits or lose consciousness*	*learning difficulty*	*Speech different from normal*	*Mentally backward or slow*	*Number*
**Age group**									
2-4	4.2	2.0	2.9	2.0	4.0	4.7	6.2	5.7	10,974
5-9	4.2	2.8	6.4	2.6	3.6	4.7	4.7	7.7	19,904
Total	4.2	2.5	5.2	2.4	3.7	4.7	5.1	6.9	30,878
**Sex**									
Male	4.5	2.5	5.2	2.5	3.8	4.9	5.6	7.4	15,280
Female	4.0	2.5	5.1	2.3	3.6	4.5	4.6	6.5	15,598
Total	4.2	2.5	5.2	2.4	3.7	4.7	5.1	6.9	30,878
**Place of residence**									
Urban	3.2	2.9	3.6	1.8	2.1	4.3	4.6	5.3	4,722
Rural	4.4	2.5	5.4	2.5	4.0	4.8	5.2	7.2	26,156
Total	4.2	2.5	5.2	2.4	3.7	4.7	5.1	6.9	30,878
**Region**									
Northern	3.3	2.3	4.0	1.8	3.0	3.5	4.5	3.6	6,133
Central	4.4	2.8	5.3	2.2	5.1	4.8	4.4	7.3	10,134
Southern	4.5	2.4	5.6	2.7	3.1	5.1	5.9	8.1	14,611
Total	4.2	2.5	5.2	2.4	3.7	4.7	5.1	6.9	30,878
**Level of Education**									
No education, Preschool	4.5	2.2	3.7	2.4	4.2	5.0	6.4	6.2	14,679
Primary	4.1	2.8	6.5	2.4	3.2	4.4	4.3	7.6	16,188

Total	4.2	2.5	5.2	2.4	3.7	4.7	5.1	6.9	30,878
**Wealth status**									
poorest	5.3	2.9	6.2	3.0	5.1	5.8	6.3	8.7	6,264
poorer	5.4	2.5	6.0	2.8	4.7	5.1	5.7	7.5	6,290
middle	4.1	2.1	5.3	2.6	3.9	4.9	4.8	7.6	6,189
richer	3.7	2.5	5.3	1.9	3.0	4.0	4.6	6.3	6,221
richest	2.7	2.5	3.0	1.6	1.8	3.7	4.1	4.6	5,914
Total	4.2	2.5	5.2	2.4	3.7	4.7	5.1	6.9	30,878
**Sex of household head**									
Male	4.3	2.4	5.1	2.3	3.6	4.6	5.1	6.7	22,131
Female	4.1	2.7	5.2	2.7	4.0	5.0	5.2	7.6	8,747
Total	4.2	2.5	5.2	2.4	3.7	4.7	5.1	6.9	30,878

Supplementary 2: Table presenting the odds of accessing assistive technology among children with disabilities in Malawi (2015-16 MDHS)

Variable	*Odds Ratio*	*Std. Err.*	*P-value*	*[95% Conf. Interval]*	
**Disability**	1.059	0.078	0.018	0.680	0.965
**Age**	1.065	0.028	0.016	1.012	1.121
**Sex**					
Male	Reference				
Female	1.100	0.172	0.541	0.810	1.494
**Place of Residence**					
Urban	Reference				
Rural	1.731	0.472	0.044	1.015	2.954
**Region**					
Northern	Reference				
Central	0.678	0.165	0.110	0.421	1.092
Southern	0.813	0.189	0.371	0.516	1.280
**Wealth status**					
poorest	Reference				
poorer	0.811	0.185	0.356	0.519	1.266
middle	0.804	0.202	0.386	0.491	1.317
richer	0.675	0.179	0.138	0.401	1.134
richest	0.968	0.227	0.890	0.611	1.533
**Sex of the Head of Household**					
male	Reference				
female	0.995	0.167	0.977	0.717	1.382
**Level of Education**					
no education, preschool	Reference				
primary	0.830	0.171	0.366	0.554	1.243
Secondary or higher	1.876	0.919	0.199	0.718	4.902
Constant	0.009	0.004	0.000	0.004	0.021

Supplementary 3: Table presenting the odds of using mobility products among children with disabilities in Malawi (2015-16 MDHS)

Variable	*Odds Ratio*	*Std. Err.*	*P-value*	*[95% Conf. Interval]*	
**Disability**	0.924	0.152	0.024	0.660	0.968
**Age**	0.976	0.057	0.677	0.870	1.094
**Sex**					
male	Reference				
female	1.112	0.425	0.781	0.526	2.350
**Residence**					
urban	Reference				
rural	3.533	1.845	0.016	1.269	9.833
**Region**					
Northern	Reference				
Central	1.453	0.838	0.517	0.470	4.499
Southern	2.236	1.202	0.134	0.780	6.412
**Level of Education**					
no education, preschool	Reference				
primary	2.567	1.380	0.080	0.895	7.365
Secondary or higher	4.140	4.794	0.220	0.428	40.048
**Wealth status**					
poorest	Reference				
poorer	0.414	0.225	0.105	0.143	1.202
middle	0.449	0.260	0.167	0.144	1.398
richer	2.173	1.498	0.260	0.563	8.393
richest	0.513	0.311	0.272	0.156	1.686
**Sex of household Head**					
Male	Reference				
Female	0.836	0.344	0.663	0.373	1.872
Constant	0.555	0.439	0.457	0.118	2.619
